# National HPV Vaccination Program in Poland—Public Awareness, Sources of Knowledge, and Willingness to Vaccinate Children against HPV

**DOI:** 10.3390/vaccines11081371

**Published:** 2023-08-16

**Authors:** Mateusz Jankowski, Justyna Grudziąż-Sękowska, Iwona Wrześniewska-Wal, Piotr Tyszko, Kuba Sękowski, Janusz Ostrowski, Mariusz Gujski, Jarosław Pinkas

**Affiliations:** 1 School of Public Health, Centre of Postgraduate Medical Education, 01-826 Warsaw, Poland; mjankowski@cmkp.edu.pl (M.J.);; 2 Department of Social Medicine and Public Health, Medical University of Warsaw, 02-007 Warsaw, Poland; 3 Institute of Rural Health in Lublin, 20-090 Lublin, Poland; 4 Department of Public Health, Medical University of Warsaw, 02-097 Warsaw, Poland

**Keywords:** HPV, HPV vaccine, human papillomavirus, vaccination strategies, cancer prevention, health policy and management, Poland

## Abstract

Since 1 June 2023, a nationwide HPV vaccination program was implemented in Poland. This study aimed to identify factors associated with public awareness of the national HPV vaccination program among adults in Poland and willingness to vaccinate children against HPV. This cross-sectional study was carried out between 14 and 17 July 2023 among 1056 adults in Poland. A self-prepared questionnaire was used. Among the respondents, 51.3% had heard about the free HPV vaccination program. The major source of knowledge on the national HPV vaccination program was TV (62%). Only 31.9% of respondents correctly indicated girls and boys aged 12 and 13 as the HPV-vaccination-eligible population. Willingness to vaccinate children against HPV was declared by 63.3% of respondents. Female gender (OR: 1.42; 95% CI: 1.11–1.81; *p* < 0.01), age 35–64 years (*p* < 0.05), having higher education (OR: 1.43; 95% CI: 1.11–1.84; *p* < 0.01), and living in cities with more than 500,000 residents (OR: 1.51; 95% CI: 1.01–2.28; *p* < 0.05) were significantly associated with higher odds to hear about the national HPV vaccination program. Age 50 years and over (*p* < 0.05), having higher education (OR: 1.43; 95% CI: 1.09–1.88; *p* < 0.05), living in cities with more than 500,000 residents (OR: 1.80; 95% CI: 1.14–2.83; *p* < 0.05), and no children under 18 in the home (OR: 1.39; 95% CI: 1.01–1.89; *p* < 0.05) were significantly associated with willingness to vaccinate children against HPV.

## 1. Introduction

Human papillomavirus (HPV) is one of the most common sexually transmitted infections [1,2]. HPV is transmitted by skin-to-skin or mucosa-to-mucosa contact [2]. There are over 200 types of human papillomaviruses [3,4]. Approximately 90% of HPV infections are asymptomatic or oligosymptomatic [4,5]. However, there are at least 14 high-risk HPV types that can cause several types of cancers. Most HPV-related cancers are attributed to HPV 16 and HPV 18 infections [5]. Infection with high-risk (oncogenic) HPV types can lead to cervical cancer, vaginal cancer, vulvar cancer, penile cancer, anal cancer, as well as cancers of the mouth, throat, or larynx [3,5,6]. Moreover, some low-risk HPV types can cause warts on/around the genitals, anus, mouth, or throat [5,7].

The most effective HPV infection prevention method is vaccination before the initiation of sexual contact [1,7]. Different public health organizations around the world issued recommendations on the prevention of new HPV infections and HPV-associated cancers and other diseases [1,8,9,10]. Usually, boys and girls aged 11–13 are HPV-vaccine-eligible populations [9,10]. However, some countries also recommend HPV vaccination for children from 9 years of age or everyone through age 26 years (if not vaccinated already) [1,5,10]. The HPV vaccine is a two-dose vaccine with a six-month interval period for children aged 9 to 15 years old [11]. Three types of HPV vaccines are licensed: 2-valent, 4-valent, and 9-valent [11].

Between 2018 and 2019, national recommendations for HPV vaccination existed in 46 of 53 (87%) WHO European Region countries [10]. However, fully or partially funded HPV vaccination was provided in 25 countries (47%) for girls and in 15 countries (28%) for boys [10]. The HPV vaccination coverage rate varied from ranged from 4.3% in Bulgaria to 99% in Turkmenistan [10]. Poland is a European Union country with a low HPV vaccination coverage rate (10–15% of the eligible population) [10,12] and a high cervical cancer burden (approximately 2500 newly diagnosed cases every year) [13,14].

In Poland, HPV voluntary vaccination was recommended for the first time in the vaccination schedule in 2008, with girls aged 11–12 years as an HPV-vaccination-eligible population [15]. As of the 2022 update of the national vaccination schedule, HPV vaccination was recommended for both girls and boys aged 9 years and over [16]. HPV vaccination was paid for out-of-pocket (approximately 30 EUR) and the vaccine was administered in primary healthcare settings [15,16,17]. However, some local government units reimbursed HPV vaccination for local populations as part of regional health policy programs [18].

Since 1 June 2023, a nationwide HPV vaccination program has been implemented in Poland [19]. The program is part of the National Oncology Strategy for 2020–2030 and was created in response to the high incidence of HPV-related cancers in Poland. All girls and boys aged 12 and 13 (born in 2010 and 2011) can benefit from free HPV vaccination [19]. The national HPV vaccination program offers free access to two types of HPV vaccines: 2-valent and 9-valent vaccines. The vaccination schedule consists of two doses, and the interval between doses of the vaccine is 6 to 12 months [19]. A parent wishing to vaccinate their child for HPV vaccination should make an appointment with a primary care practice providing vaccinations within the program. A visit for vaccination can be arranged directly at the facility, via the central helpline, or using an e-health tool called Patient’s Internet Account [19,20]. Health communication strategy for the national HPV vaccination program included media campaigns (TV, radio), billboards, and leaflets as well as web marketing (including social media) [19]. By 14 July 2023, over 56 thousand children aged 12–13 years were vaccinated against HPV within the program [19]. Based on the number of live births between 2010 and 2011, the HPV-vaccination-eligible population can be estimated at 800,000 children [21]. The current HPV vaccination coverage rate within the national HPV vaccination program can be estimated at 7% of the eligible population.

There is a lack of scientific data on public awareness of the national HPV vaccination program as well as exposure to health communication campaigns on HPV vaccination and sources of knowledge on HPV vaccination. Most of the previously published data on HPV vaccination in Poland were carried out before the introduction of the universal and free HPV vaccination for children aged 12 and 13 [17,22,23,24,25]. Identification of the factors associated with public awareness of the national HPV vaccination program, eligible populations, and attitudes toward vaccinating children may significantly contribute to the better promotion of the program and may increase the HPV vaccination coverage rate in Poland.

Therefore, this study aimed to identify factors associated with public awareness of the national HPV vaccination program among adults in Poland and the willingness to vaccinate children against HPV, one month after the introduction of the program.

## 2. Materials and Methods

### 2.1. Study Design and Population

This cross-sectional survey is based on nationwide representative data on public awareness of the national HPV vaccination program in Poland launched on 1 June 2023. Data were collected between 14 and 17 July 2023 among 1056 adults in Poland. A computer-assisted web interviewing (CAWI) technique was used. Data were collected by a public opinion research company [26] using the protocol prepared by the authors.

The public opinion research company selected respondents from over 100,000 users of the web-based research platform. The study population was selected based on the non-probability quota sampling methods [21,26]. The stratification model included gender, age, and place of residence (along with regional distribution throughout Poland). The study sample was representative of the general population of Poland [21]. Similar methods were used in previous nationwide cross-sectional surveys in Poland [20,27].

This study was approved by the Ethics Committee at the Centre of Postgraduate Medical Education in Warsaw (approval number 307/2023 of 12 July 2023).

### 2.2. Measures

The study questionnaire included five questions on public awareness of the national HPV vaccination program, sources of knowledge on the program, willingness to vaccinate children against the HPV, HPV-vaccine-eligible populations as well as the oncogenic potential of HPV.

*Awareness of the national HPV vaccination program:* “Have you ever heard about the free HPV vaccination program implemented as part of the National Cancer Strategy 2020–2030?” (yes/no). Those who said yes were asked to indicate the source of information about the HPV vaccination program (multiple choice question—ten different sources of information). Moreover, all respondents were asked about the HPV-vaccination-eligible population (within the program).

*Willingness to vaccinate children against HPV:* “Would you like to vaccinate your child against human papillomavirus (HPV)?” with a 5-point Likert scale. Respondents who said “definitely yes” or “rather yes” were considered as those who declared willingness to vaccinate children against HPV.

*The oncogenic potential of HPV:* “What do you think are HPV-related diseases? Cervical cancer; vaginal cancer; penile cancer; cancer of the mouth, throat or larynx; laryngeal papillomatosis; anal cancer; genital warts”, each disease with three possible answers: yes/no/I do not know.

*Sociodemographic variables:* Financial status was self-reported (good/moderate/bad), as most of the respondents denied indicating the income per family member. Occupational status was defined based on the question: “What is your current professional status?” (active occupational status: currently employed or self-employed; passive active occupational status: currently unemployed/pensioner/student). Respondents who indicated bachelor’s degree or completed higher education were defined as those having higher education. The study questionnaire is presented in Supplementary Material S1.

### 2.3. Statistics

SPSS version 28 (IBM, Armonk, NY, USA) was used to analyze the data. Frequencies and proportions were used to present the distribution of categorical variables. To compare categorical variables, cross-tabulations, and chi-squared tests were performed. Binary Logistic Regression in SPSS was used to assess the relationships between sociodemographic characteristics (nine different variables) and selected attitudes towards HPV vaccination (separated models: (1) awareness of the national HPV vaccination program; (2) awareness of HPV-vaccination-eligible population; (3) willingness to vaccinate children against HPV; and (4) awareness of the oncogenic potential of HPV). In bivariable analysis, all variables were considered independently. In multivariable regression models, only statistically significant variables in bivariable analysis were included. The odds ratio (OR) and 95% confidence intervals (95% CI) were used to assess the strength of the associations. The statistical significance level was based on the criterion *p* < 0.05.

## 3. Results

Data were received from 1056 respondents aged 18–83 years, 53.2% were females. The characteristics of the study population are presented in Table 1.

### 3.1. Public Awareness of the National HPV Vaccination Program

Among the respondents, 51.3% had heard about the free HPV vaccination program implemented as part of the National Cancer Strategy 2020–2030. TV (62%) was the major source of knowledge on the national HPV vaccination program. Moreover, more than one-fourth of respondents indicated information on the news portal (23.1%), Internet advertising (20.5%), and radio (20.5%) as the source of knowledge on the national HPV vaccination program (Table 2). Only 31.9% of respondents correctly indicated girls and boys aged 12 and 13 as a target population that can be vaccinated within the national HPV vaccination program. Willingness to vaccinate (“definitely yes” or “rather yes”) children against HPV was declared by 63.3% of respondents (Table 2). Only 60.1% of respondents declared that HPV infection can cause cervical cancer, 51.6% were aware that HPV can lead to vaginal cancer and 42.1% of respondents indicated that HPV could cause penile cancer. Less than one-third of respondents believed that HPV infection can cause cancer of the mouth, throat, or larynx (Table 2).

### 3.2. Sociodemographic Differences in the Public Awareness of the National HPV Vaccination Program

Females more often (*p* < 0.05) declared that they had heard about the national HPV vaccination program. The percentage of respondents who were aware of the HPV vaccination program, eligible population, and declared willingness to vaccinate a child against HPV increased (*p* < 0.05) with age (Table 3). Respondents with higher education more often (*p* < 0.05) declared that they had heard about the national HPV vaccination program and were aware of the eligible population and declared willingness to vaccinate a child against HPV. Those who were married more often (*p* < 0.05) declared the correct populations eligible for the HPV vaccination program. Respondents who had children more often (*p* < 0.05) declared that they had heard about the national HPV vaccination program and were aware of the eligible population and declared willingness to vaccinate a child against HPV. Respondents with passive occupational status (pensioners/students/unemployed) more often (*p* < 0.05) declared willingness to vaccinate children against HPV. Moreover, respondents with a bad financial status of the family more often (*p* < 0.05) declared willingness to vaccinate their child against HPV (Table 3).

### 3.3. Sources of Knowledge on HPV Vaccination Program by Sociodemographic Variables

The percentage of respondents (Table 4) who heard about the national HPV vaccination program from TV or news portal increased with age (*p* < 0.05). The percentage of respondents who heard about the national HPV vaccination program was the highest among the youngest respondents (*p* < 0.05). Respondents with higher education more often indicated radio and news portals as a source of knowledge on the national HPV vaccination program, but less often indicated TV (Table 4). Those who had children more often indicated radio and news portals as sources of knowledge about the national HPV vaccination program (*p* < 0.05). Those who did not have children at home more often (*p* < 0.05) indicated TV and doctors as sources of knowledge about the national HPV vaccination program. Occupationally passive respondents more often (*p* < 0.05) indicated TV as a source of knowledge about the national HPV vaccination program (Table 4).

### 3.4. Factors Associated with Public Awareness of the National HPV Vaccination Program

Female gender (OR: 1.42; 95% CI: 1.11–1.81; *p* < 0.01), age 35–64 years (*p* < 0.05), having higher education (OR: 1.43; 95% CI: 1.11–1.84; *p* < 0.01), and living in cities above 500,000 residents (OR: 1.51; 95% CI: 1.01–2.28; *p* < 0.05) were significantly associated with higher odds of hearing about the national HPV vaccination program (Table 5). Age 35 years and over was significantly associated (*p* < 0.05) with higher odds of correctly indicating HPV-vaccination-eligible population (girls and boys aged 12 and 13). Age 50 years and over (*p* < 0.05), having higher education (OR: 1.43; 95% CI: 1.09–1.88; *p* < 0.05), living in cities with more than 500,000 residents (OR: 1.80; 95% CI: 1.14–2.83; *p* < 0.05), and no children under 18 in the home (OR: 1.39; 95% CI: 1.01–1.89; *p* < 0.05) were significantly associated with willingness to vaccinate child against HPV (Table 5).

## 4. Discussion

This is the first study to assess the public awareness of the national HPV vaccination program launched in Poland on 1 June 2023. All girls and boys aged 12 and 13 (born in 2010 and 2011) can benefit from free HPV vaccination. The program was widely promoted in traditional media as well as the Internet [19]. Findings from this study revealed that only 51.3% of adults in Poland had heard of the national HPV vaccination program and 31.9% were aware of the HPV-vaccination-eligible population. TV was the most common source of knowledge on the national HPV vaccination program, wherein Internet and social media were indicated by less than one quarter of respondents. Willingness to vaccinate children against HPV was declared by 63.3% of respondents. Out of nine sociodemographic factors, age, educational level, place of residence, and having children were significantly associated with willingness to vaccinate children against HPV.

In Poland, HPV vaccination has been listed in the vaccination schedule by the public health authorities as voluntary vaccination since 2008 [15]. Before the implementation of the national HPV vaccination program, HPV vaccinations were promoted mostly by healthcare professionals and public health authorities [15,22,24]. Smolarczyk et al. showed that in 2018, only 62.5% of parents in Poland were aware of HPV vaccination [22]. Drejza et al. showed that in 2019, 58.2% of young adults in Poland were not aware of their HPV vaccination status [23]. In 2021, Sypień and Zielonka showed that only 54% of Polish parents had heard about HPV [24]. Most of the studies on HPV vaccination in Poland were carried out on non-representative samples [22,23,24].

The abovementioned studies indicated the low level of public awareness of HPV vaccination and its role in cancer prevention [22,23,24]. In this study, 51.3% of adults had heard of the national HPV vaccination program that was launched in June 2023. The HPV vaccination program was widely promoted on TV, radio, press as well as the Internet and social media [19]. Moreover, news portals regularly published information on HPV vaccination programs and their rules. Despite the widespread media campaign, only half of adults in Poland were aware of the national HPV vaccination program. In media campaigns, HPV vaccination was promoted as a cervical cancer prevention method [19]. Findings from this study showed that cervical cancer was the most recognized HPV-related cancer, indicated by 60.3% of respondents. This observation is in line with the media campaigns on HPV vaccinations that are focused on cervical cancer prevention [28]. Both HPV vaccines in Poland are effective in the prevention of genital cancers, so educational campaigns should also include HPV-related cancers other than cervical cancer [2,5]. As boys are eligible for HPV vaccination, more attention should be paid to penile cancer and genital warts as potential consequences of HPV infection [5]. In 2021, in a representative sample of adults in Poland, Pinkas et al. showed that only 32.5% of adults Poland indicated HPV infection as a risk factor for head and neck cancer [17]. In this study, 32.3% of adults in Poland indicated that HPV infection can lead to cancer of the mouth, throat, or larynx. This observation suggests that despite the implementation of the national HPV vaccination program, the percentage of adults in Poland who are aware of the oncogenic potential of HPV infection (other than cervical cancer) remains unchanged [17].

Smolarczyk et al. [22], Sypień and Zielonka [24], and Pinkas et al. [17] showed that higher education is significantly associated with a higher level of awareness of HPV and HPV vaccination. In this study, females, adults aged 35–64 years, respondents with higher education, and those living in the largest cities had higher odds of hearing the national HPV vaccination program. Findings from this study suggest, that current communication campaigns on HPV vaccination programs are mostly targeted to well-educated individuals from large cities in Poland. Further media campaigns should target parents without higher education, who live in rural areas and small/medium-sized cities. Sypień and Zielonka [24] showed that conversations with a doctor are strong motivators to vaccinate children against HPV, so healthcare professionals, especially those in rural areas should be actively involved in the promotion of HPV vaccination programs. To increase the engagement of healthcare professionals [29], a system of rewards for achieving the vaccination coverage rate of the eligible population (e.g., 75–80%) should be considered. In this study, females were more aware of the HPV vaccination program, which may result from the fact, that HPV vaccination was promoted as a cervical cancer prevention method. We can hypothesize, that adults aged 35–64 years are those who had children aged 12–13 years (eligible population), so they are more aware of the HPV vaccination program.

In Poland, girls and boys aged 12 and 13 are eligible for free HPV vaccination [19]. Despite the fact that 51.3% of adults in Poland had heard of the national HPV vaccination program, only 31.9% of all respondents correctly indicated the HPV-vaccination-eligible population. This observation suggests that most of the adults in Poland only heard of the HPV vaccination program, but did not know the details, e.g., eligible populations. More attention should be paid to informing about the HPV-vaccination-eligible populations [10,29]. Moreover, we can hypothesize that parents of children aged over 13 years may be interested in HPV vaccination after the media campaigns on HPV vaccination, so public health authorities should also communicate how children aged 14 and over can be vaccinated against HPV, even within additionally paid (out-of-pocket) services. Out of nine sociodemographic factors, only the age 35 years and over were significantly associated with higher odds of awareness of HPV-vaccination-eligible populations. This observation suggests that personalized communication is needed to target parents of children aged 12 and 13 years.

The HPV vaccination coverage rate in Poland is estimated at 7–15% of the eligible population [19,21]. By 14 July 2023 (the first six weeks of the program) approximately 7% of the eligible population was vaccinated within the program [19]. In this study, 63.3% of respondents declared willingness to vaccinate children against HPV. This percentage is significantly higher than reported by Pinkas et al., in 2021 when 48.1% of adults in Poland (a nationwide representative sample, similar to this study) declared willingness to vaccinate children against HPV [17]. This observation suggests that the introduction of the national HPV vaccination program, accompanied by media campaigns [30] led to an increase in public trust in HPV vaccination and vaccine confidence levels among adults in Poland. Age 50 years and over, higher education, and living in cities with over 500,000 residents were significantly associated with higher odds of declaring willingness to vaccinate children against HPV. In this study, people without higher education who live in rural areas and small/medium size cities were also less likely to hear about the national HPV vaccination program. We can hypothesize that lower acceptance of the HPV vaccine among those without higher education who live in rural areas and small/medium size cities may result from the fact that they were not informed about the HPV vaccination program [30,31]. In this study, people with bad financial status were more likely to declare willingness to vaccinate children against HPV. This observation requires further investigation.

*Practical implications:* this is the first analysis of the national HPV vaccination program implemented in Poland since June 2023. Findings from this study revealed that TV is a major source of knowledge on the HPV vaccination program, and more actions are needed to promote this program in social media. Moreover, this study revealed that there were no sociodemographic differences in the exposure to HPV-related social media campaigns, which suggests that personalized communication was not used. Moreover, this study indicated that only one third of adults in Poland are aware of the HPV-vaccination-eligible population, which may lead to misunderstanding and lack of understanding of the patient’s path. This study also showed that most of the adults in Poland were not aware of HPV-related diseases, e.g., penile cancer, anal cancer, and cancer of the mouth, throat, or larynx. Most of the promotional campaigns on the HPV vaccination program in Poland were focused on cervical cancer [23,24], so more actions are needed to communicate HPV-related cancers that may be diagnosed also in boys (e.g., anal cancer or cancer of mouth, throat, or larynx).

*Limitations:* this study was carried out on a nationwide representative sample of adults in Poland, so people without children were also included. Further studies should be carried out among parents of children aged 12 and 13 years (HPV-vaccination-eligible population). The list of sources of knowledge on the HPV vaccination program was limited to the most common ones. Willingness to vaccinate children against HPV was self-declared and medical documentation was not verified (declaration vs. vaccination decision).

## 5. Conclusions

This study showed that one-third of the adults in Poland have never heard of the national HPV vaccination program. The HPV vaccine is well-accepted by the public, but most of the respondents were not aware of the HPV-vaccination-eligible population despite the implementation of the informative campaign on the HPV vaccination program. The communication strategy for the HPV vaccination program should include personalized communication on social media addressed to parents of children eligible for HPV vaccination. General practitioners should be more involved in promoting HPV vaccination. Education on HPV-related cancers, other than cervical cancer should be included in media campaigns. Lessons learned from the implementation of the national HPV vaccination program in Poland can be used by other countries during the implementation of national HPV vaccination programs.

## Figures and Tables

**Table 1:** Characteristics of the study population (n = 1056).

Variable	n	%
Gender		
Female	562	53.2
Male	494	46.8
Age [years]		
18–34	329	31.2
35–49	277	26.2
50–64	235	22.3
65+	215	20.4
Higher education		
Yes	459	43.5
No	597	56.5
Married		
Yes	575	54.5
No	481	45.5
Place of residence		
Rural area	382	36.2
City <20,000 inhabitants	137	13.0
City ≥20,000–99,999 inhabitants	215	20.4
City ≥100,000–499,999 inhabitants	186	17.6
City ≥500,000 inhabitants	136	12.9
Having children		
Yes	701	66.4
No	355	33.6
Number of children		
0	242	22.9
1	459	43.5
2 or more	355	33.6
Children <18 years in home		
Yes	343	32.5
No	713	67.5
Occupational activity		
Active	617	58.4
Passive	439	41.6
Financial status of the family		
Good	210	19.9
Moderate	379	35.9
Bad	467	44.2

**Table 2:** Public awareness of the national HPV vaccination program (n = 1056).

Variable	n	%
Have you ever heard about the free HPV vaccination program implemented as part of the National Cancer Strategy 2020–2030?		
Yes	542	51.3
No	514	48.7
Please indicate all sources from which you heard about the national HPV vaccination program? (n = 542)		
TV	336	62.0
Radio	111	20.5
Printed press/newspapers	49	9.0
Doctor	66	12.2
Nurse	43	7.9
Poster/flyer in a medical facility	63	11.6
Internet advertising	111	20.5
Social media	104	19.2
Information on the news portals	125	23.1
Other source of knowledge	24	4.4
Please indicate the HPV-vaccination-eligible population		
Only girls under 18	45	4.3
Girls and boys under 18 years of age	160	15.2
Only girls aged 12 and 13	83	7.9
Girls and boys aged 12 and 13	337	31.9
I do not know	431	40.8
Would you like to vaccinate your child against HPV?		
Definitely no	68	6.4
Rather no	78	7.4
Rather yes	310	29.4
Definitely yes	358	33.9
I do not know	242	22.9
What do you think are HPV-related diseases?		
Cervical cancer	635	60.1
Vaginal cancer	545	51.6
Penile cancer	445	42.1
Anal cancer	357	33.8
Cancer of the mouth, throat or larynx	341	32.3
Laryngeal papillomatosis	355	33.6
Genital warts	510	48.3

**Table 3:** Public awareness of the national HPV vaccination program by sociodemographic factors (n = 1056).

Variable	Have You Ever Heard About the National HPV Vaccination Program			Correctly Indicated HPV-Vaccination-Eligible Population			Willingness to Vaccinate Child Against HPV		
	n	%	*p*	n	%	*p*	n	%	*p*
Gender									
Female	312	55.5	**0.004**	189	33.6	0.2	349	62.1	0.4
Male	230	46.6		148	30.0		319	64.6	
Age [years]									
18–34	139	42.2	**0.001**	66	20.1	**<0.001**	178	54.1	**<0.001**
35–49	156	56.3		98	35.4		166	59.9	
50–64	130	55.3		86	36.6		153	65.1	
65+	117	54.4		87	40.5		171	79.5	
Higher education									
Yes	263	57.3	**<0.001**	164	35.7	**0.02**	320	69.7	**<0.001**
No	279	46.7		173	29.0		348	58.3	
Married									
Yes	307	53.4	0.1	201	35.0	**0.02**	375	65.2	0.1
No	235	48.9		136	28.3		293	60.9	
Place of residence									
Rural area	183	47.9	0.2	116	30.4	0.08	216	56.5	**0.004**
City <20,000 inhabitants	71	51.8		36	26.3		90	65.7	
City ≥20,000–99,999 inhabitants	111	51.6		75	34.9		141	65.6	
City ≥100,000–499,999 inhabitants	95	51.1		55	29.6		120	64.5	
City ≥500,000 inhabitants	82	60.3		55	40.4		101	74.3	
Having children									
Yes	381	54.4	**0.01**	242	34.5	**0.01**	455	64.9	0.1
Yo	161	45.4		95	26.8		213	60.0	
Number of children									
0	161	45.4	**0.01**	95	26.8	**0.02**	213	60.0	0.3
1	124	51.2		78	35.7		160	66.1	
2 or more	257	56.0		164	35.7		295	64.3	
Children <18 years in home									
Yes	184	53.6	0.3	97	28.3	0.08	186	54.2	**<0.001**
No	358	50.2		240	33.7		482	67.6	
Occupational activity									
Active	306	49.6	0.2	178	28.8	**0.01**	375	60.8	**0.04**
Passive	236	53.8		159	36.2		293	66.7	
Financial status of the family									
Good	104	49.5	0.4	62	29.5	0.1	113	53.8	**0.002**
Moderate	188	49.6		110	29.0		238	62.8	
Bad	250	53.5		165	35.3		317	67.9	

**Table 4:** Exposure to HPV-related health communication campaigns by sociodemographic factors and source of information (n = 542).

	Sources from Which Respondents Heard about the National HPV Vaccination Program														
Variable	TV			Radio			Doctor			Information on the News Portals			Social Media		
	n	%	*p*	n	%	*p*	n	%	*p*	n	%	*p*	n	%	*p*
Gender															
Female	192	61.5	0.8	55	17.6	0.06	42	13.5	0.3	67	21.5	0.3	57	18.3	0.5
Male	144	62.6		56	24.3		24	10.4		58	25.2		47	20.4	
Age [years]															
18–34	64	46.0	**<0.001**	23	16.5	0.3	26	18.7	**0.02**	21	15.1	**0.01**	26	18.7	0.8
35–49	92	59.0		38	24.4		17	10.9		34	21.8		27	17.3	
50–64	89	68.5		24	18.5		16	12.3		32	24.6		25	19.2	
65+	91	77.8		26	22.2		7	6.0		38	32.5		26	22.2	
Higher education															
Yes	150	57.0	**0.02**	67	25.5	**0.01**	28	10.6	0.3	77	29.3	**<0.001**	45	17.1	0.2
No	186	66.7		44	15.8		38	13.6		48	17.2		59	21.1	
Married															
Yes	197	64.2	0.2	75	24.4	**0.01**	36	11.7	0.7	77	25.1	0.2	62	20.2	0.5
No	139	59.1		36	15.3		30	12.8		48	20.4		42	17.9	
Place of residence															
Rural area	115	62.8	0.8	35	19.1	0.3	23	12.6	0.2	36	19.7	0.3	35	19.1	0.9
City <20,000 inhabitants	48	67.6		15	21.1		3	4.2		12	16.9		13	18.3	
City ≥20,000–99,999 inhabitants	68	61.3		26	23.4		15	13.5		29	26.1		19	17.1	
City ≥100,000–499,999 inhabitants	56	58.9		24	25.3		16	16.8		25	26.3		22	23.2	
City ≥500,000 inhabitants	49	59.8		11	13.4		9	11.0		23	28.0		15	18.3	
Having children															
Yes	244	64.0	0.1	88	23.1	**0.02**	46	12.1	0.9	97	25.5	**0.04**	76	19.9	0.5
No	92	57.1		23	14.3		20	12.4		28	17.4		28	17.4	
Number of children															
0	92	57.1	0.2	23	14.3	0.06	20	12.4	0.06	28	17.4	0.1	28	17.4	0.2
1	75	60.5		31	25.0		22	17.7		32	25.8		31	25.0	
2 or more	169	65.8		57	22.2		24	9.3		65	25.3		45	17.5	
Children <18 years in home															
Yes	94	51.1	**<0.001**	39	21.2	0.8	30	16.3	**0.04**	44	23.9	0.7	36	19.6	0.9
No	242	67.6		72	20.1		36	10.1		81	22.6		68	19.0	
Occupational activity															
Active	175	57.2	**0.01**	71	23.2	0.07	41	13.4	0.3	69	22.5	0.7	64	20.9	0.2
Passive	161	68.2		40	16.9		25	10.6		56	23.7		40	16.9	
Financial status of the family															
Good	64	61.5	0.5	18	17.3	0.07	16	15.4	0.4	23	22.1	0.5	19	18.3	0.8
Moderate	111	59.0		31	16.5		24	12.8		39	20.7		39	20.7	
Bad	161	64.4		62	24.8		26	10.4		63	25.2		46	18.4	

**Table 5:** Factors associated with public awareness of the national HPV vaccination program (n = 1056).

	Factors Associated with Public Awareness of the National HPV Vaccination Program					
Variable	Have You Ever Heard about the National HPV Vaccination Program		Correctly Indicated HPV-Vaccination-Eligible Population (Girls and Boys Aged 12 and 13)		Willingness to Vaccinate Child against HPV	
	Bivariable Logistic Regression	Multivariable Logistic Regression	Bivariable Logistic Regression	Multivariable Logistic Regression	Bivariable Logistic Regression	Multivariable Logistic Regression
	OR (95% CI)	OR (95% CI)	OR (95% CI)	OR (95% CI)	OR (95% CI)	OR (95% CI)
Gender						
Female	1.43 (1.12–1.83) **	1.42 (1.11–1.81) **	1.19 (0.91–1.54)		0.90 (0.70–1.16)	
Male	Reference	Reference	Reference		Reference	
Age [years]						
18–34	Reference	Reference	Reference	Reference	Reference	Reference
35–49	1.76 (1.28–2.43) ***	1.61 (1.13–2.29) **	2.18 (1.51–3.14) ***	2.22 (1.49–3.30) ***	1.27 (0.92–1.75)	1.36 (0.97–1.92)
50–64	1.69 (1.21–2.37) **	1.51 (1.03–2.20) *	2.30 (1.58–3.36) ***	2.24 (1.46–3.43) ***	1.58 (1.12–2.24) **	1.50 (1.04–2.15) *
65+	1.63 (1.15–2.31) **	1.41 (0.95–2.10)	2.71 (1.85–3.97) ***	2.27 (1.39–3.71) ***	3.30 (2.22–4.90) **	3.00 (1.90–4.76) ***
Higher education						
Yes	1.53 (1.20–1.95) ***	1.43 (1.11–1.84) **	1.36 (1.05–1.77) *	1.29 (0.98–1.70)	1.65 (1.27–2.13) ***	1.43 (1.09–1.88) *
No	Reference	Reference	Reference	Reference	Reference	Reference
Married						
Yes	1.20 (0.94–1.53)		1.36 (1.05–1.77) *	1.11 (0.80–1.53)	1.20 (0.94–1.55)	
No	Reference		Reference	Reference	Reference	
Place of residence						
Rural area	Reference	Reference	Reference	Reference	Reference	Reference
City <20,000 inhabitants	1.17 (0.79–1.73)	1.17 (0.78–1.73)	0.82 (0.53–1.27)	0.82 (0.53–1.29)	1.47 (0.98–2.21)	1.46 (0.96–2.23)
City ≥20,000–99,999 inhabitants	1.16 (0.83–1.62)	1.14 (0.81–1.60)	1.23 (0.86–1.75)	1.23 (0.86–1.78)	1.46 (1.04–2.07) *	1.42 (0.99–2.03)
City ≥100,000–499,999 inhabitants	1.14 (0.80–1.61)	1.08 (0.75–1.54)	0.96 (0.66–1.41)	0.95 (0.64–1.41)	1.40 (0.97–2.01)	1.32 (0.91–1.93)
City ≥500,000 inhabitants	1.65 (1.11–2.46) **	1.51 (1.01–2.28) *	1.56 (1.04–2.34) *	1.49 (0.97–2.27)	2.22 (1.44–3.42) ***	1.80 (1.14–2.83) *
Having children						
Yes	1.44 (1.11–1.85) **	1.19 (0.88–1.62)	1.44 (1.09–1.91) *	0.91 (0.63–1.32)	1.23 (0.95–1.60)	
No	Reference	Reference	Reference	Reference	Reference	
Children <18 years in home						
Yes	1.15 (0.89–1.49)		0.88 (0.59–1.04)		Reference	Reference
No	Reference		Reference		1.76 (1.35–2.29) ***	1.39 (1.01–1.89) *
Occupational activity						
Active	0.85 (0.66–1.08)		Reference	Reference	Reference	Reference
Passive	Reference		1.40 (1.08–1.82) *	1.29 (0.93–1.80)	1.30 (1.00–1.67) *	0.95 (0.69–1.30)
Financial status of the family						
Good	Reference		Reference		Reference	Reference
Moderate	1.00 (0.72–1.41)		0.98 (0.67–1.41)		1.45 (1.03–2.04) *	1.40 (0.98–2.00)
Bad	1.17 (0.85–1.63)		1.30 (0.92–1.85)		1.81 (1.30–2.53) ***	1.75 (1.23–2.49) **

* *p* < 0.05; ** *p* < 0.01; *** *p* < 0.001.

## Data Availability

Data are available from the corresponding author on reasonable request.

## Supplementary Materials

The following supporting information can be downloaded at: https://www.mdpi.com/article/10.3390/vaccines11081371/s1, Supplementary Material S1: Study questionnaire.
