# Supplementary material for: National HPV Vaccination Program in Poland—Public Awareness, Sources of Knowledge, and Willingness to Vaccinate Children against HPV
Source: Vaccines (Basel). 2023 Aug 16;11(8):1371. doi: 10.3390/vaccines11081371 (PMC10459249; doi:10.3390/vaccines11081371)
Supplement: Supplementary file 1 [file vaccines-11-01371-s001.zip › vaccines-2543915-supplementary.pdf]

Study Questionnaire – HPV vaccination programme in Poland

Read the description below, then answer the questions.

HPV (Human Papilloma Virus) is the name of the human papilloma virus. The HPV virus is responsible for some cancers, and one of the methods of prevention is vaccination.

[Q1] Have you ever heard about the free HPV vaccination program implemented as part of the National Cancer Strategy 2020-2030?
Yes
No

[if heard] **[Q2] Please indicate all sources from which you heard about the national HPV vaccination program?** [multiple choice question]
TV
radio
printed press/newspapers
doctor
nurse
poster/flyer in a medical facility
Internet advertising
social media
information on the news portals
other source of knowledge

[Q3] Please indicate the HPV-vaccination-eligible population
only girls under 18
girls and boys under 18 years of age
only girls aged 12 and 13
girls and boys aged 12 and 13
I don't know

[Q4] Would you like to vaccinate your child against HPV?
definitely no
rather no
rather yes
definitely yes
I don't know

[Q5] What do you think are HPV-related diseases?

LP	Diease	Yes	No	I do not know
1	cervical cancer			
2	breast cancer			
3	vaginal cancer			
4	penile cancer			
5	anal cancer			
6	cancer of the mouth, throat or larynx			
7	laryngeal papillomatosis			
8	genital warts			

Metrics

1. What is your gender?
woman
man

2. What age are you?
18-24 years
25-34 years old
35-44 years
45-54 years
55 years or older

Please indicare your year of age_________

3. Where do you live? Select the size of the town where you live
Rural area
small town (up to 20,000 inhabitants)
medium-sized city (from 20 to 99 thousand inhabitants)
big city (from 100 to 500 thousand inhabitants)
big city (over 500,000 inhabitants)

4. What is your current education (last school)?
primary or secondary school
essential
medium
post-secondary or post-secondary
bachelor degree
completed higher education

5. How do you generally self-assess the current financial situation of your family?
bad
hard to say, some bad and some good (moderate)
good

6. What is your current professional status?
I work on a contract of employment
I work on civil contract
I am working on a bussines contract
I run my own business
unemployed
retiree or pensioner
pupil or student
I run the house
other (enter which)

7. What is your marital status?
free (single)
married
in an informal relationship
other (enter which)

8. Do you have children?
Yes (Please indicate how many __)
No

9. Do children under 18 live with you?
Yes
No
